# Factors Influencing the Adoption of Voluntary Nonpharmaceutical Interventions to Control COVID-19 in Japan: Cross-sectional Study

**DOI:** 10.2196/34268

**Published:** 2022-08-02

**Authors:** Makoto Kuroki, Kiyoshi Yamamoto, Shaun Goldfinch

**Affiliations:** 1 School of Economics and Business Administration Yokohama City University Yokohama Japan; 2 Kamakura Women's University Kamakura Japan; 3 University of Tokyo Tokyo Japan; 4 Australia New Zealand School of Government Melbourne Australia; 5 Curtin University Perth Australia

**Keywords:** COVID-19, nonpharmaceutical interventions, social distancing, phone tracing, trust in government, confidence in scientists

## Abstract

**Background:**

Trust in government is seen to facilitate crisis management and policy instrument adoption across numerous studies. However, in Japan, public support for government handling of the COVID-19 pandemic and trust in the government is low, yet the adoption of voluntary nondigital nonpharmaceutical interventions (NPIs) is high. This is an important tension this study seeks to unravel.

**Objective:**

The aim of this study is to understand the antecedents of nondigital NPI and tracking app adoption in the COVID-19 pandemic in Japan.

**Methods:**

A commercial company was contracted to deliver an online survey of 1248 Japanese citizens in December 2020. A quota technique was used to deliver a sample representative in terms of gender, age, residence, income, and education.

**Results:**

The adoption of voluntary nondigital NPIs is predicted by confidence in public health scientists and a favoring of infection control over reducing economic and social costs. A novel and unexpected finding is that trust in government does not predict nondigital NPI use. Perceived risk and knowledge of infection did not increase the use of nondigital NPIs. Education and income were not significant factors, although female and older respondents demonstrated greater compliance. For the adoption of a phone tracking app, trust in government is important, as is urban residence, albeit with a lower use of the app compared to nondigital NPIs.

**Conclusions:**

Voluntary compliance in the adoption of nondigital NPIs—if skillfully led by trusted scientific experts and in accord with societal norms—can be effectively achieved. We provide evidence that trust in government is effective in encouraging the use of the Japanese tracking app. Moreover, the technical efficacy of digital initiatives and perceptions of such will unsurprisingly affect citizen support and use of digital tools.

## Introduction

### Background

A body of literature suggests that trust in government facilitates crisis management and citizen compliance with government directives and suasion during the COVID-19 pandemic [1]. Moreover, trust in government initially increased in several countries, partly due to perceptions of effective pandemic management [2]. Trust in government remains low in Japan, however [3]. Moreover, despite the relatively low death toll, surveys show low levels of support for the Japanese government’s handling of the pandemic. Indeed, according to the Pew Research Center [4], only 55% of Japanese respondents believed that the government had dealt properly with the COVID-19 pandemic, a portion lower than that of Italy (74%) and Sweden (71%), both of which recorded more COVID-19 deaths as a proportion of the population. However, compliance with voluntary nondigital nonpharmaceutical interventions (NPIs) has been high, as this study will show. Hence, the role of trust in government in Japan in the adoption of NPIs is likely to be more complex than other studies suggest.

Apart from vaccination, the Japanese government recommended a range of NPIs, including hand washing, face mask wearing, social distancing, staying at home, and the use of ventilation. Citizens were encouraged to avoid the so-called 3Cs: closed spaces, crowded places, and close-contact settings. A contact-tracing app, the COVID-19 Contact-Confirming Application (COCOA), was developed and launched by the government on June 16, 2020. In most cases, there was no penalty for flouting government directives due in part to constitutional constraints prioritizing individual privacy and freedom. Instead, the adoption of NPIs depended on voluntary citizen compliance with government guidelines, which was largely achieved, albeit with lower contract-tracing app use compared to other NPIs. This is in marked contrast to some other countries’ use of coercive and legal means to enforce lockdowns and other NPI adoption [5,6].

This study seeks to clarify the factors associated with NPI and tracing app adoption. Drawing on an online representative survey of 1248 Japanese citizens and testing 3 groups of hypotheses, we show that the adoption of nondigital NPIs is predicted by confidence in public health scientists and support for infection control over economic and social costs. Trust in government does not predict nondigital NPI use—an unexpected and counterintuitive finding. Perceived risk or knowledge of infection does not increase nondigital NPI use. Education and income are not significant factors although females and older respondents demonstrate greater compliance. Regarding the adoption of a phone-tracking app, trust in government is important, as is urban residence, but confidence in public health scientists is not.

### Hypotheses

A variety of NPIs have been used worldwide to control the pandemic including social distancing, mask wearing, lockdowns, staying at home, hand washing, ventilation, and the adoption of digital tracing. Border closures, curfews, and other measures could also be classified as NPIs. There are historical precedents for NPI use, including in the Spanish flu and SARS (severe acute respiratory syndrome) epidemics, and quarantining and social distancing have long histories in previous pandemics. There is also recent empirical support for their efficacy. For example, Bo et al [7] examined the effectiveness of mandatory mask use, isolation or quarantine, social distancing, and traffic restrictions; they confirmed significant reduction in morbidity across the 190 countries studied. Haug et al [8] analyzed the impact of various NPIs and found support for their effectiveness. China, the United States, the United Kingdom, and the European nations have used contact tracing through digital means, which are claimed to be effective [9-12].

Trust in government may predict NPI adoption during a pandemic [13]. A large body of research suggests trust in government is associated with policy instrument adoption [1,2]. For example, Goldfinch et al [2] found that trust in government and confidence in public health scientists predicted phone-tracking app use in Australia and New Zealand. Studies in the United States found that trust in government and government sources was associated with adoption of social distancing [14]. Moreover, with citizens relying on experts’ guidance, confidence in scientific expertise is likely to be related to the adoption of NPIs. A multinational survey company, Ipsos, reported that doctors and scientists are trusted more than are governments by citizens worldwide [15]. In the case of Japan, (medical) doctors are rated “the first most trustworthy” (52%) and scientists are rated “the second most trustworthy” (43%) [15]. Kazemian et al [16] found in the United States that scientific trust raised support (although adoption was not measured) for COVID-19 social-distancing policies [16]. Given the Japanese context, we focus on the 6 NPIs recommended by the government: wearing facemasks, washing hands, social distancing, refraining from going out, avoiding the 3Cs, and maintaining ventilation. This wide range of measures adds to the novelty of this study.

This discussion leads us to our first set of hypotheses: hypothesis 1a—trust in government is associated with compliance with preventive behavior directives; hypothesis 1b—confidence in public health scientists is associated with compliance with preventive behavior directives.

Perceived risk, fear, and knowledge of the disease might be a factor in NPI adoption [6]. For example, Pedersen and Favero’s [5] online survey of US residents found willingness to maintain social distance was predicted by perceived risk of the pandemic. Harper et al’s [17] UK study found that a leading antecedent for adopting NPIs was the fear of COVID-19. Webster et al’s [18] review found factors affecting adherence to protective health behaviors included levels of knowledge about the disease outbreak and risk of disease. Moreover, NPIs can have marked economic effects, and aspects of social isolation involved with staying at home and social distancing have profound psychological impacts, which are yet to be fully determined [19,20]. The decision to adopt NPIs then may also be an act of balancing one perceived risk—that is, the disease—against the risk of economic and social disruptions. Moreover, individuals may be more likely to accept new and unorthodox measures if the perceived loss from not doing so is greater, so how relative risks are framed by them and others will likely affect behavior [21,22]. Hence, an individual’s attitude toward the trade-offs of pandemic control may affect NPI compliance and contact-tracing app use. From this discussion, we develop the following hypotheses: hypothesis 2a—compliance with NPI requirements is higher when risk perception (knowledge) is higher; hypothesis 2b—compliance with NPI requirements is higher when infection control is prioritized over economic and social effects; hypothesis 2c—contact-tracing app use is higher when infection control is prioritized over economic and social effects; hypothesis 2d—contact-tracing app use is higher when risk perception (knowledge) is higher.

In the case of digital tracing, there are added concerns about privacy of information that is shared with and gathered by the government although the Japanese contact-tracing app does not collect personal information. Hence, it deserves dedicated study. Chan and Saqib’s [23] experiments suggest that the reluctance to download contact-tracing apps can be explained by privacy concerns. Horvath et al’s [24] experimental study suggests that trust in the UK National Health Service mitigates respondents’ concerns about privacy in tracing apps, with the benefit of using tracing apps perceived to be larger than the risks of privacy and security breaches. Oldeweme et al [25] found that transparency and trust in government foster tracking app adoption. Goldfinch et al [2] found that in Australia and New Zealand, trust in government and confidence in public health scientists predict the use of contact-tracing apps [2]. Trust in government then provides a proxy for addressing privacy and security concerns that might arise with digital engagement. Accordingly, we propose the following hypotheses: hypothesis 3a—contact-tracing app use is higher when trust in the government is higher; hypothesis 3b—contact-tracing app use is higher when confidence in public health scientists is higher.

## Methods

### Survey

A commercial company was contracted to deliver an online survey of 1248 Japanese citizens in December 2020. A quota technique was used to deliver a sample representative in terms of gender, age, residence, income, and education. Respondents included both men and women in the age groups of 18-24, 25-34, 35-44, 45-54, 55-64, and 65 years and above. Quota methods are widely used in public health, medical, and epidemiological research and are considered state of the art [26,27].

### Variables

The dependent variables are compliance with preventative behavior (CPB) and use of the contact-tracing app (COCOA).

CPB was measured via the following question: “To what extent do you comply with the government recommended behaviors for protection from COVID-19?” The 6 nondigital NPIs recommended by the Japanese government are the following: (1) wearing face masks, (2) hand washing, (3) social distancing, (4) refraining from going out, (5) avoiding the 3Cs, and (6) ventilation.

Responses were given on a 4-point Likert scale ranging from 4 = “always” to 1 = “not at all.” As the responses might have had different weights, we performed principal component analysis and used a single principal component score. Although there are several methods for determining the number of factors, we adopted the most representative and easily understood eigenvalue-1 criterion [28].

Use of the contact-tracing app (COCOA) was measured by the response to the following question: “To what extent do you use COCOA?” Responses were on a 4-point Likert scale ranging from 4 = “always” to 1 = “not at all.”

The independent variables were operationalized as follows. Trust in government (Trust government) was measured based on the response to the statement, “(level of government) is generally trustworthy.” The response was marked on a 4-point Likert scale ranging from 4 = “strongly agree” to 1 = “strongly disagree.” This was derived from Goldfinch et al [2]. The answers for the national and local governments were summed. Confidence in scientific expertise (Confidence expertise) was determined by asking, “How much do you believe that public health scientists act in the best interests of the public?”, which was also derived from Goldfinch et al [2]. Responses were marked on a 4-point Likert scale ranging from 4 = “a great deal” to 1 = “not at all.”

We measured the perceived risk of infection (knowledge) by loosely adapting questions from Wise et al [29]. The perceived risk of COVID-19 (Perception risk) was measured via responses to the following 3 questions: “Have you ever received any information that your families are infected?”, “Have you ever received any information that your coworkers are infected?”, and “Have you ever received any information that other related persons such as clients are infected?” The answers were 1 = yes and 0 = no. The answers were summed. Previous studies have inquired about hypothetical “average” people (eg, the average person in the neighborhood, state, and country). As the Japanese media’s focus was on the number of cases in the country, we focused on the extent to which family members, coworkers, and others had information about the infected rather than specifying the geographic area.

Respondents’ perceptions of the appropriate balance between infection control and society and economy (Economics) were derived from the following question: “To which element do you attach more importance: infection control or maintenance of economic and social activities?” Responses were on a 4-point Likert scale ranging from 1 = “emphasize infection control more than economic and social activities” to 4 = “emphasize economic and social activities more than infection control.” This question was derived by the authors based on concerns about balancing economic performance with infection risk, which is often discussed in Japan.

Sociodemographic factors may also influence NPI compliance. Riou et al [30] found adoption of protective behaviors to be correlated with age and comorbidity risk in China. Females are more likely to comply with government directives in general [31]. Older people might feel less at ease using contact-tracing apps that demand some digital competence but may be more compliant with government directives [32]. Accordingly, our other independent variables were the following: gender (male = 1 or female = 0), age (1 = 18-24, 2 = 25-34, 3 = 35-44, 4 = 45-54, 5 = 55-64, and 6 = 65 years and above), education (from 1 = secondary school graduate to 5 = postgraduate), residence (urban = 1, rural= 0), and household income (1 = up to ¥2 million, 2 = ¥2-3 million, 3 = ¥3-4 million, 4 = ¥4-6 million, 5 = ¥6-8 million, 6 = ¥8-10 million, 7 = ¥10-12 million, 8 = ¥12-15 million, and 9 = ¥15 million and above; a currency exchange rate of ¥1=US $0.007 is applicable).

### Statistical Analysis

Adoption of NPIs (CPB) was analyzed using the ordinary least squares multiple regression model, as shown in equation 1. Use of the contract-tracing app (COCOA) was analyzed via an ordered logistic regression model by maximum likelihood estimation, as seen in equation 2. The estimated coefficients explain the change in log odds of using COCOA. Analysis was performed using Stata/IC 16.1 (StataCorp).

CPB = α_1_ + α_2_Trust government+ α_3_Confidence expertise + α_4_Perceptionrisk + α_5_Economics + α_6_Gender + α_7_Age + α_8_Income + α_9_Education + α_10_Urban + ε_1_
**(1)**

COCOA = β_1_ + β_2_Trust government + β_3_Confidence expertise + β_4_Perceptionrisk + β_5_Economics + β_6_Gender + β_7_Age + β_8_Income + β_9_Education + β_10_Urban + ε_2_
**(2)**

Here, the dependent and independent variables *CPB*, *COCOA*, *Trust government*, *Confidence expertise*, *Perceptionrisk*, and *Economics* are calculated as shown in the previous subsection. *Gender* is a dummy variable which is 1 if a respondent is a man, 0 otherwise. The variables *Age*, *Income*, and *Education* are continuous variables from responses. *Urban* is a dummy variable which is 1 if a respondent answers as living in an urban area, 0 otherwise.

### Ethical Considerations

Ethics approval was obtained from the chairman of the Committee for Assessing Ethics on Research at Kamakura Women’s University. The survey company received full confirmation from the survey monitors to consent with its privacy policy and quality control. The final contract for the survey was approved by the president of the university (application #110853).

## Results

### Descriptive Statistics

Reported adoption of (nondigital) NPIs was high. Approximately 80% of respondents said they “always” or “mostly” behave according to government guidelines, with 97.20% (1213/1248) wearing masks, 95.43% (1191/1248) washing hands, 85.98% (1073/1248) engaging in social distancing, 78.21% (976/1248) refraining from going out, 83.25% (1039/1248) avoiding the 3Cs, and 76.52% (955/1248) using ventilation (see Table 1). The use of COCOA was considerably lower, with 57.85% (722/1248) nonusage. Trust in government in Japan was alarmingly low, with strong agreement or “agreement that government are generally trustworthy” at 1.60% (20/1248) and 35.74% (446/1248), respectively—far lower than that of other OECD (Organization for Economic Co-operation and Development) countries [2]. This finding of low trust in government in Japan is supported by other studies [3]. Confidence in public health scientists was far greater: 42.15% (526/1248) of respondents had “a great deal” of confidence and 48.32% (603/1248) were “fairly” confident that they work in the public interest.

Table 2 presents the respondents’ demographic characteristics. Gender and age were manipulated to have the same percentages during the online survey phase. Annual household income was most frequently between ¥6 and ¥8 million yen, and approximately 46% of the respondents had a college degree or higher. This sample is broadly representative although respondents had a lower rate of higher education and slightly higher income than Japan has as a whole [33,34]. 

Table 3 shows the Pearson correlation coefficients among the variables. The correlation coefficients between CPB and COCOA and the independent variables were consistent with the results of the regression analysis. All correlation coefficients were lower than 0.3; hence, the potential for multicollinearity was small.

**Table 1:** Compliance with nonpharmaceutical interventions and COCOA (N=1248).

	Always, n (%)	Mostly, n (%)	Little, n (%)	Not at all, n (%)
Wearing mask	962 (77.08)	251 (20.11)	28 (2.24)	7 (0.56)
Washing hands	832 (66.67)	359 (28.77)	51 (4.09)	6 (0.48)
Social distancing	380 (30.45)	693 (55.53)	158 (12.66)	17 (1.36)
Refraining from going out	354 (28.37)	622 (49.84)	231 (18.51)	41 (3.29)
Avoiding 3Cs^a^	370 (29.65)	669 (53.61)	189 (15.14)	20 (1.60)
Ventilation	343 (27.48)	612 (49.04)	271 (21.71)	22 (1.76)
Using contact-tracing apps (COCOA^b^)	214 (17.15)	126 (10.10)	186 (14.90)	722 (57.85)

^a^3Cs: closed spaces, crowded places, and close contacts.

^b^COVID-19 Contact-Confirming Application.

**Table 2:** Demographic statistics (N=1248).

		Values
**Gender, n (%)**		
	Men	624 (50)
	Women	624 (50)
**Age (years), n (%)**		
	18-24	208 (16.7)
	25-34	208 (16.7)
	35-44	208 (16.7)
	45-54	208 (16.7)
	55-64	208 (16.7)
	65 or more	208 (16.7)
Age, mean (SD)		45.0 (16.8)
**Residence, n (%)**		
	Rural area	666 (53.37)
	Urban area	582 (46.63)
**Income (yen^a^), n (%)**		
	<2 million	80 (6.40)
	<2 to 3 million	252 (20.20)
	3 to <4 million	226 (18.10)
	4 to <6 million	519 (41.59)
	6 to <8 million	92 (7.40)
	8 to <10 million	38 (3.00)
	10 to <12 million	29 (2.30)
	12 to <15 million	8 (0.60)
	>15 million	4 (0.30)
Income, mean (SD)		3.1 (1.3)
**Education, n (%)**		
	Junior high school graduate	25 (2.00)
	High school graduate	364 (29.17)
	College graduate	273 (21.88)
	University graduate	539 (43.19)
	Graduate degree	47 (3.77)

^a^A currency exchange rate of ¥1=US $0.007 is applicable.

**Table 3:** Pearson correlation matrix.

	Variable	1, *r*	2, *r*	3, *r*	4, *r*	5, *r*	6, *r*	7, *r*	8, *r*	9, *r*	10, *r*	11, *r*
1	CPB^a^	—^b^										
2	COCOA^c^	0.214	—									
3	Trust in government	0.043	0.073	—								
4	Confidence expertise	0.206	0.073	0.176	—							
5	Perception risk	–0.047	0.026	–0.018	0.073	—						
6	Economics	–0.220	–0.010	0.055	–0.087	0.005	—					
7	Gender	–0.142	0.058	0.052	–0.065	0.051	0.120	—				
8	Age	0.087	–0.044	0.041	–0.029	–0.138	–0.150	0.007	—			
9	Income	–0.035	0.045	0.021	–0.011	0.126	0.077	0.076	0.045	—		
10	Education	0.014	0.056	0.063	0.006	0.116	0.013	0.191	–0.056	0.167	—	
11	Urban	0.026	0.053	0.003	0.020	0.067	0.044	0.000	–0.004	0.082	0.087	—

^a^CPB: compliance with preventative behavior.

^b^Not applicable.

^c^COCOA: COVID-19 Contact-Confirming Application.

### Hypothesis Testing

Testing our hypotheses provided unexpected, counterintuitive, and novel results (Table 4). First, trust in government was not significantly associated with the adoption of NPIs. Hence, hypothesis 1a was not supported. Confidence in public health scientists had a positive and significant effect on NPI adoption. Therefore, hypothesis 1b was supported. However, risk perception or knowledge had a negative and significant relationship with preventative behavior. This was an unexpected and counterintuitive finding. Consequently, hypothesis 2a was rejected. As expected, a belief that greater attention should be paid to economic effects rather than to infection (Economics) had a negative and significant relation with NPI adoption, and thus hypothesis 2b was supported. This is consistent with other findings that framing COVID-19 as primarily a health issue promotes a preference for social distancing, whereas economic framing motivates the opposite [33]. Men (Gender) were less likely to comply with government recommendations, consistent with previous findings that women are more law abiding [31]. Older people (Age) were more likely to adopt recommended NPIs, in line with studies finding a positive relationship between age and a law-abiding orientation [32]. Income (Income), education (Education), and residence (Urban) were not significantly associated with preventative behaviors.

To test hypotheses 2c-2d and 3a-b examining tracing app use (COCOA), an ordered logit analysis was implemented. The results, shown in Table 5, are not always as predicted. Trust in government (Trust government) had a positive and significant effect on the use of COCOA, supporting hypothesis 3a. However, in contrast to NPI adoption, confidence in scientific expertise (Confidence expertise) did not have a significant impact on using the contact-tracing app. As a result, hypothesis 3b was not supported. Attitudes toward the economic or infection trade-off (Economics) also did not have a significant effect on the use of the app. Hence, hypothesis 2c was not supported. Risk (Perception risk) was also nonsignificant, and hypothesis 2d was not supported. Additionally, no other independent variable, except residence (Urban), had a significant effect on using the app. However, people living in urban areas (Urban) were more likely to use the contact-tracing app compared to those living in rural areas.

**Table 4:** Regression results for CPB (N=1248)^a^.

Variable	Predicted sign	CPB^b^ values			
		*r*	*t*	*P* value	95% CI
Constant	N/A^c^	–1.879	–4.494	<.001^d^	–2.700 to –1.059
Trust government	+	0.056	0.689	.46	–0.104 to 0.216
Confidence expertise	+	0.484	6.017	<.001^d^	0.326 to 0.642
Perception risk	+	–0.140	–1.880	.07^e^	–0.286 to 0.006
Economics	–	–0.899	–5.802	.<.001^d^	–1.203 to –0.595
Gender	–	–0.403	–4.108	.<.001^e^	–0.596 to –0.211
Age	+	0.006	2.185	.03^f^	0.001 to 0.012
Income	Unknown	–0.022	–0.580	.54	–0.097 to 0.053
Education	Unknown	0.082	1.518	.11	0.053 to 0.188
Urban	Unknown	0.109	1.156	.25	–0.076 to 0.295

^a^The table shows the results estimated using the ordinary least squares regression model in equation 1.

^b^CPB: compliance with preventative behavior.

^c^N/A: not applicable.

^d^Statistical significance at the 1% level.

^e^Statistical significance at the 10% level.

^f^Statistical significance at the 5% level.

**Table 5:** Ordered logistic regression results for COCOA (N=1248)^a^.

Variable	Predicted sign	COCOA^b^ values			
		*r*	*t*	*P* value	95% CI
Constant 1	N/A^c^	1.385	3.332	<.001^d^	0.560 to 2.208
Constant 2	N/A	2.059	4.965	<.001^d^	1.235 to 2.880
Constant 3	N/A	2.661	6.455	<.001^d^	1.841 to 3.476
Trust government	+	0.247	2.616	.009^d^	0.063 to 0.433
Confidence expertise	+	0.107	1.233	.22	–0.064 to 0.276
Perception risk	+	–0.139	–0.869	.41	–0.447 to 0.179
Economics	–	–0.005	–0.055	.60	–0.191 to 0.187
Gender	–	0.177	1.560	.11	–0.040 to 0.405
Age	+	–0.006	–1.647	.097^e^	–0.012 to 0.001
Income	Unknown	0.049	1.126	.35	–0.049 to 0.131
Education	Unknown	0.030	0.504	.59	–0.086 to 0.150
Urban	Unknown	0.181	1.648	.097^e^	–0.038 to 0.402

^a^The table shows the results estimated using the ordered logistic regression model in equation 2.

^b^COCOA: COVID-19 Contact-Confirming Application.

^c^N/A: not applicable.

^d^Statistical significance at the 1% level.

^e^Statistical significance at the 10% level.

## Discussion

### Principal Findings

The use of NPIs to control the COVID-19 spread will remain important as the global vaccination rollout progresses unevenly and new COVID-19 variants emerge. Moreover, the lessons from this pandemic will assist the management of the next one, if or when it arises. Japan provides a particularly useful field to examine NPI use and its antecedents, particularly because it relies on voluntary adoption rather than on the compulsory use and legal and other sanctions adopted by other OECD countries. Despite the voluntary nature of NPI adoption, respondents from our representative online quota sample reported high use of the 6 types of nondigital NPIs we measured in the 80% and above range. This is in remarkable contrast to the claimed 30%-40% noncompliance rates for use of NPIs in the United States and the European Union despite legal enforcement and significant penalties for noncompliance [5,6]. Moreover, trust in government—seen as a predictor of policy compliance in several studies—is low in Japan and does not predict the use of nondigital NPIs.

### Comparison With Prior Work

How can these differences be explained? Drawing on the limited but growing literature on the adoption of NPIs, we tested hypotheses focused on trust in government, confidence in public health scientists, perceived trade-offs between infection control and socioeconomic effects, and perceptions of infection risk based on knowledge of infection among close contacts. Our results sometimes differ from those predicted or indicated by other studies, and, therefore, this study reports a few novel, unexpected, and counterintuitive findings. First, trust in government did not have a significant effect on NPI use. Attention to personal hygiene is often claimed to be an existing aspect of Japanese culture [35]. Moreover, readiness to adopt preventative behaviors in disease control, such as mask wearing, is commonly exhibited. Trust in government may have little or no relationship to this. The second and more perplexing finding is that respondents’ risk perception inversely affected NPI adoption or was not significant in terms of phone app use. Our risk measure is a perception based on the reported knowledge of infection among those close to the respondents. Wise et al [29] also reported that risk perception or infection knowledge did not increase concerns about COVID-19 morbidity [29]. Perhaps perceived risk to oneself is not a key driver of NPI adoption or other COVID-19–related control behavior in Japan. Rather, prosocial motivation, social conformity, and cultural constraints may be more important [5,36-39].

Confidence in public health scientists predicts compliance with government NPI directives. Preventive behavior was recommended by a group of experts on COVID-19, and it was associated with scientific leadership rather than political. A generalizable point is that confidence in scientific expertise can encourage compliance with health policy even when trust in government is low. In the past, the Japanese public has demonstrated the capability to be guided by trusted experts in new control measures—particularly when information and responses are clearly articulated and communicated—and to dramatically change behavior as a result [40]. This has been exhibited during the recent pandemic. In April 2020, Hayasaki [41] quoted a Japanese professor in political science, Koichi Nakano, noting “People remain largely ignorant of the basic principles of ‘social distancing’—a term that remains unknown and alien in Japan.” However, within a few months, social distancing became part of Japanese life. Moreover, this adaptation has been seen across educational, residential, and income categories. These new behaviors are locked in place, and compliance maintained perhaps more through cultural conformity and control than through government fiat, with Japanese culture categorized as collectivist and a premium being placed on compliance with societal norms and group solidarity [36-38]. Once the control measures are successfully signaled by trusted scientific authorities and adopted by a significant portion of society, there arises a social risk of not complying [42-45].

Trust in government and urban residence predicts tracking app use (COCOA). Confidence in public health scientific expertise, however, was not significantly associated with the use of the app. Moreover, reported use of the app was lower than that of other NPIs. This may reflect the troubled development of the app. There may be a disjunction between public scientific expertise related to disease control and an app developed by a contracted nongovernment commercial operation plagued by bugs and low reliability. In collectivist Japan, the less easily observable phone app use may lack the “virtue signaling” of other forms of NPI, such as mask wearing, and hence be less enforceable by societal norms and censure. Trust, privacy protection, and technical efficacy, as well as perceptions of such, are likely to be important in the citizen adoption of digital tools for public health in the future. People living in urban areas are more likely to adopt the contact app, which might be related to a more densely populated environment, with a higher number of contacts and a higher chance of infection. However, risk perception does not have a significant effect on the use of COCOA.

### Limitations

Our study has a few limitations. First, the analysis was cross-sectional, and behavior, including adoption of NPIs, might change as fatigue and complacency sets in. To examine causal relations and behavioral changes over time, a longitudinal study would perhaps be useful. A better operationalization of the antecedents of NPI and COCOA adoption, such as personality and cultural factors, might improve the verisimilitude of our results. Our use of an online survey method might have excluded those marginalized by a “digital divide,” particularly important in COVID-19, which may produce unequal outcomes based on socioeconomic status. However, in a time of lockdowns, travel bans, and other controls, an online survey was likely the most pragmatic solution. Generalizability would be improved with further cross-national studies, including an investigation of institutional and cultural factors. Other studies using panel data, alternative methods of sample selection (including nondigital ones), experiments, new antecedents, qualitative interviews, and comparative studies might strengthen findings, by, for example, providing a better understanding of causal relationships.

### Conclusions

What are the implications for the implementation and development of public health and health policies? First, we show that voluntary compliance in the adoption of nondigital NPIs—if skillfully led by trusted scientific experts and in accord with societal norms—can be effectively achieved. Despite the voluntary nature of NPI adoption, respondents reported high use of the NPI we measured in the 80% and above range. This contrasts with the 30%-40% noncompliance rates for use of NPIs in the United States and the European Union despite legal enforcement and significant penalties for noncompliance [5,6]. Second, digitalization in the public sector should balance trade-offs between perceived usefulness and privacy. This may be resolved if trust in government can be developed and maintained, and we provide evidence that trust in government is effective in encouraging the use of digital government services at least in the case of the COCOA tracking app. Moreover, technical efficacy of digital initiatives and perceptions of such will unsurprisingly affect citizen support and use of digital tools. Perhaps this is generalizable to the adoption of other digital tools and e-government in policy and public health, in which Japan remains a laggard. Risk perception and how risk is framed and focused around social and health outcomes may improve NPI uptake, again underpinning the importance of clear and focused communication in developing support and citizen compliance in pandemic control found in other studies [46].

## Abbreviations

3Cs: closed spaces, crowded places, and close-contact settings
COCOA: COVID-19 Contact-Confirming Application
CPB: compliance with preventative behavior
NPI: nonpharmaceutical intervention
OECD: Organization for Economic Co-operation and Development
SARS: severe acute respiratory syndrome
